# Pathogenesis and management of gastrointestinal inflammation and fibrosis: from inflammatory bowel diseases to endoscopic surgery

**DOI:** 10.1186/s41232-021-00174-7

**Published:** 2021-07-14

**Authors:** Kentaro Iwata, Yohei Mikami, Motohiko Kato, Naohisa Yahagi, Takanori Kanai

**Affiliations:** 1 Division of Gastroenterology and Hepatology, Department of Internal Medicine, Keio University School of Medicine, Tokyo, Japan; 2 Division of Research and Development for Minimally Invasive Treatment, Cancer Center, Keio University School of Medicine, Tokyo, Japan

**Keywords:** Gastrointestinal fibrosis, Crohn’s disease, Endoscopic surgery

## Abstract

Gastrointestinal fibrosis is a state of accumulated biological entropy caused by a dysregulated tissue repair response. Acute or chronic inflammation in the gastrointestinal tract, including inflammatory bowel disease, particularly Crohn’s disease, induces fibrosis and strictures, which often require surgical or endoscopic intervention. Recent technical advances in endoscopic surgical techniques raise the possibility of gastrointestinal stricture after an extended resection. Compared to recent progress in controlling inflammation, our understanding of the pathogenesis of gastrointestinal fibrosis is limited, which requires the development of prevention and treatment strategies. Here, we focus on gastrointestinal fibrosis in Crohn’s disease and post-endoscopic submucosal dissection (ESD) stricture, and we review the relevant literature.

## Background

Gastrointestinal stricture is the pathological thickening of the wall of the gastrointestinal tract, characterized by excessive accumulation of extracellular matrix (ECM) and expansion of the population of mesenchymal cells. Gastrointestinal stricture leads to blockage of the gastrointestinal tract, which significantly reduces a patient’s quality of life. Upper gastrointestinal stricture may cause nausea, vomiting, anorexia, and abdominal pain because of food stagnation. In addition to these obstructive symptoms, lower gastrointestinal stricture may cause intestinal perforation, intra-abdominal abscess, and fistulizing disease because of increased pressure in the region of the inflamed intestinal tract.

Malignant and benign processes cause gastrointestinal stricture as well as inflammation and the healing of surgical wounds. Fibrostenosis of the gastrointestinal tract, in particular, is a frequent complication of Crohn’s disease. Further, a recent highly significant advance in endoscopic treatment enables resection of premalignant and early-stage gastrointestinal cancers. This procedure does not involve surgical reconstruction of the gastrointestinal tract, although fibrotic stricture after endoscopic treatment is an emerging clinical problem. Here, we focus on post-endoscopic scarring and Crohn’s disease, which cause artificial and spontaneous fibrosis of the gastrointestinal tract, and we review shared and unique mechanisms of pathogenesis.

## Current endoscopic treatment and challenges

Endoscopic mucosal resection (EMR) and ESD are endoscopic techniques for resecting epithelial tumours with low risk of metastasis. EMR is a conventional method to resect relatively small and superficial tumours. A metal ring (named snare forceps) is used to capture the lesion that is excised using a high-frequency electric current. EMR is simple and safe; however, it is limited to relatively small (e.g. less than approximately 20-mm diameter) lesions.

ESD was first reported in 1999 by Gotoda et al. [1]. Unlike EMR, ESD enables secure resection, regardless of lesion size or location, through precise dissection of the submucosal layer. The costs of ESD of the stomach were initially covered by health insurance in Japan in 2006, in 2008 for the oesophagus, and in 2011 for the colon. With the widespread use of screening endoscopy, the chances of early detection of cancer have increased [2–4], and it is a standard treatment worldwide. Moreover, when applied to gastric cancer, ESD achieves higher en-bloc resection rates and lower local recurrence [5, 6] for oesophageal [7] and duodenal cancers [7].

**Fig. 1:**
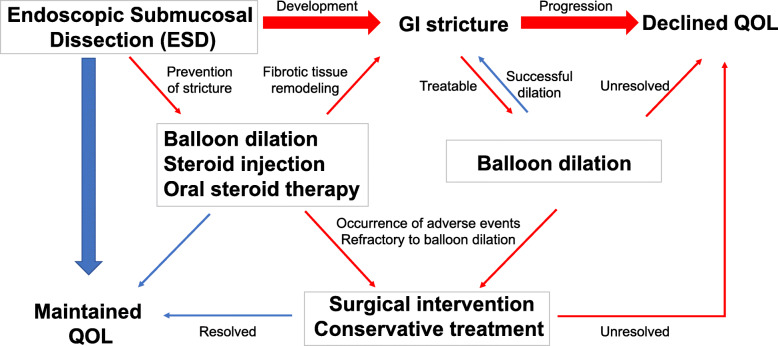
Management of fibrostenosis after ESD. Prophylactic balloon dilatation, localized steroid injection therapy, and oral steroid therapy are administered to prevent fibrostenosis after ESD. If a GI stricture develops, balloon dilation or surgical treatment is considered. Multiple types of treatment lead to diminution of a patient’s QOL

An advantage of ESD is its ability to securely resect lesions independent of their size; however, fibrostenosis may occur after extended resection as a relatively frequent late adverse event (Fig. 1). In particular, an issue in clinical practice, oesophageal stricture associated with submucosal fibrosis often develops during the healing of post-ESD ulcers, extending to approximately 75% of the circumference [8–10], whereas the clinical impact of post-ESD stricture is relatively less in the stomach, duodenum, and colorectum compared to oesophagus. There are some possible factors causing this different susceptibility for post-ESD stricture depending on the organs. First, the lumen of the oesophagus is narrow, and undigested food passes through it; therefore, even relatively mild stricture can easily cause symptoms such as dysphagia. Second, in the rectum and duodenum, which are anatomically fixed to the retroperitoneum, even large mucosal defects are less likely to cause stricture [11]. Third, stricture could be prevented by approximating the wounds along longitudinal direction; however, this is not possible in the oesophagus because of the lack of excess of mucosa. After endoscopic surgery in the oesophagus, the oesophageal lumen is narrow so that the wounds frequently contact each other or ingested food and liquid, which evokes subsequent infiltration of immune cells and production of chemokines and cytokines. These inflammatory responses in the healing process direct centre-directed healing and result in the oesophageal stricture. Recent attempts of “tissue-shielding therapy” such as transplantation of oral mucosal cell sheets and polyglycolic acid sheets aim to cover the wound surface and prevent the mechanical contact of ingested substances or the neighbouring side of the wound surface [12]. These novel methods show some promising preliminary results but have not yet achieved complete protection of oesophageal stricture after endoscopic surgery. The postoperative oesophageal stricture leads to decreased quality of life, characterized as dysphagia and vomiting, even if the cancer or dysplasia is successfully removed. Methods such as prophylactic balloon dilatation, locoregional steroid injection therapy, and oral steroid therapy effectively prevent oesophageal stricture [13]. However, oesophageal lacerations and bleeding occur as complications of balloon dilatation [7] and delayed perforation of steroidal injection [14], which may require surgery.

Recently, implantation of oral mucosal epithelial cell sheets [15], PGA-felt and fibrin gluing [16], and biodegradable stents [17], although useful, are not employed in routine clinical practice because of their cost, time required, and technical problems. Further, although tissue biopsy is important for definitive diagnosis before administering ESD, submucosal fibrosis often develops after biopsy. Unfortunately, progress in increasing our understanding of the pathogenesis of gastrointestinal fibrosis and efforts to develop prevention and treatment methods lag behind the advances in ESD technology.

## Healing of oesophageal ulcers after endoscopy

After endoscopic treatment, fibrosis terminates in 4 weeks, and infiltration of inflammatory cells occurs in the submucosa 2–4 days after the creation of a so-called “artificial ulcer”. After 7 days, epithelial cells proliferate, the number of inflammatory cells in the submucosal tissue decreases, and fibrous tissue associated with angiogenesis proliferates. After 28 days, fibrous tissue replaces the lesion. In the oesophagus, oesophageal glands or mucosal fascia are not observed after the completion of epithelialization of the artificial ulcer, and the epithelium and submucosal layer are thinner than usual [18].

Nonaka et al. [19] found that spindle-shaped myofibroblasts, which express α-SMA, are present in the base of the ulcer 1 week after the creation of an artificial ulcer, which contributes to the formation of the stricture. In contrast, spindle-shaped myofibroblasts are irregularly located in the fibrous region of the repaired tissue after topical steroid injection for prophylaxis of oesophageal stricture. Further, keratinocyte growth factor (KGF), hepatocyte growth factor (HGF), prostaglandin E-prostanoid 2 receptors, cAMP, and cAMP response element-binding protein contribute to the repair of the oesophageal epithelium [19–22].

However, few reports employ animal models of oesophageal fibrostenosis compared with those of the small and large intestines. This is partially attributed to the technical difficulties involved in approaching the oesophagus of a small animal. Further, the lack of suitable transgenic animals (e.g. mice) makes it difficult to determine the contributions of certain cell subsets, cytokines, chemokines, growth factors, and other effectors. Available models of fibrostenosis of the oesophagus, such as those employing the 100% acetic acid-induced oesophagitis model in Sprague-Dawley rats [23], and post-ESD oesophageal ulceration model in pigs [24] and dogs [25]. Thus, small animal models of stable oesophageal fibrostenosis help identify the mechanism of pathogenesis of oesophageal fibrosis.

## Clinical features and epidemiology of chronic inflammatory conditions that cause strictures in the gastrointestinal tract

Benign oesophageal strictures are caused by different aetiologies. Gastroesophageal reflux disease and eosinophilic gastritis have been two major causes of the oesophageal strictures, but recent technological advances in cancerous treatment strategies including radiation and endoscopic surgery highlight the rapid increase in iatrogenic or secondary strictures after treatment [26]. It is of note that most of the aetiologies are associated with the inflammatory process followed by stenosis and it is important to understand both inflammatory and remodelling phages of the gastrointestinal tract. Among the multiple aetiologies of oesophageal strictures, Crohn’s disease is a rare but important condition known to cause strictures in the small and large intestines [26, 27]. We here summarize the aetiology and pathologies of Crohn’s disease because it is one of the fields where the fibrosis mechanisms have been extensively studied and the knowledge in the Crohn’s strictures may be shared across the entire gastrointestinal tract. The incidence of Crohn’s disease in Japan shows a clear, recently increasing trend. Crohn’s disease is characterized by chronic granulomatous inflammation that may involve any region of the gastrointestinal tract, predominantly the terminal ileum and adjacent colon, and presents with a segmental, asymmetric distribution [28, 29]. The main symptoms are abdominal pain, diarrhoea, fistula, anal lesions, and systemic symptoms differing in severity [30]. Crohn’s disease frequently manifests extraintestinal complications such as nodular erythema, necrotizing pyoderma, polymorphic exudative erythema, iritis, and vaginitis [31]. Recurrence of this progressive disease leads to major complications. Despite recent advances in treatment, these intra- and extra-intestinal complications impair the quality of life.

Crohn’s disease is characterized by discontinuous skip lesions that are observed during endoscopy [32]. Intestinal stricture is a common complication of Crohn’s disease, affecting approximately 33% of patients within 10 years of onset. Treatment of Crohn’s disease aims to achieve sustained clinical and endoscopic remission (“low entropy”) and to interrupt the naturally progressive destructive disease course that culminates in intestinal failure and associated complications (“high entropy”). Although multiple clinical, environmental, serological, genetic, and epigenetic markers are potential predictors of fibrostenotic Crohn’s disease (Table 1) [33–35], we lack specific and reliable markers that represent the state of gut fibrosis and predict stricturing.

**Table 1:** Predictors of fibrostenosing Crohn’s disease

Clinical	Age at diagnosis < 40 years
	Perianal disease at diagnosis
	Need for steroids during first flare
	Early use of azathioprine or anti-TNF
	Small bowel disease location
	Prior appendectomy
Environmental	Smoking
Endoscopic	Deep mucosal laceration
Genetic	Janus-associated kinase 2 (*JAK2*)
	*ATG16L1*
	*NOD2/CARD15* mutations on both chromosomes
	TNF superfamily 15 (*TNFSF15*) in Asians
	5T5T in the *MMP3* gene
	rs1363670
Serological	Antimicrobial antibodies
	Anti-*Saccharomyces cerevisiae* (ASCA) IgA in Asians

Behçet’s disease, sometimes referred to as the “Silk Route disease” because of its elevated frequencies in the Middle East and far-eastern Asia [36–38] which are traditionally considered endemic areas. HLA-B51 is a risk factor for Behçet’s disease [36, 37]. The most pronounced symptoms of Behçet’s disease are associated with the intestine. Intestinal Behçet’s disease typically forms a round to oval swell-like ulcer in the terminal ileum. Ulcers may form in the entire gastrointestinal tract, although oesophageal lesions are infrequent [39]. Cases of intestinal Behçet’s disease with an aphthous ulcer may be difficult to differentiate from Crohn’s disease because the morphology of the former is similar to that of early Crohn’s disease. In addition, Crohn’s disease and Behçet’s disease can both affect the entire gastrointestinal tract and cause ulcers because of chronic autoimmune inflammation.

The differences in endoscopic morphology between these otherwise similar diseases include the characteristics of the ulcer base, depth, shape, and margin. A solitary deep oval ulcer with a thick exudative necrotic layer at the ulcer bottom serves as a signature of intestinal Behçet’s disease compared with the ulcer of Crohn’s disease. Further, plasma cells in the granulation tissue of Behçet’s disease accumulate to abnormally high levels. Inflammation surrounding the ulcer margin and ulcer bed is milder and more localized than in Crohn’s disease. Epitheloid granuloma is detected in approximately 50% of patients with Crohn’s disease who undergo surgical resection of the intestine. Moreover, focal cryptitis, basal plasmacytosis, lymphoid aggregates, and nerve fibre hyperplasia are detected in Crohn’s disease.

## Pathophysiology of Crohn’s disease

Gastrointestinal bacterial species among healthy individuals are diverse, and this diversity may be significantly influenced by dietary and drug-induced factors. Dysbiosis is involved in the onset and exacerbation [40] of Crohn’s disease [41, 42]. The diversity of bacterial species representing the phyla *Firmicutes* and *Bacteroidetes* is reduced in patients with Crohn’s disease [43–45]. However, a recent large-scale multiomics analysis conducted as a component of the Integrative Human Microbiome Project (HMP2) found that metagenomic species differ significantly between patients with Crohn’s disease and controls [46]. These features of dysbiosis remain to be established as causes or consequences of Crohn’s disease.

Previous studies mainly focus on the microbiota of the faeces, which widely differs from that of the small intestine. More recent studies employing endoscopy of the small bowel show that the microbiome of the mucosal tissues of the small intestine harbours several bacterial species that are closely associated with Crohn’s disease [47].

The small intestine is covered with a single layer of a simple columnar epithelium. Goblet cells are present in the intestinal villi and secret a mucus biofilm to protect the mucosa [43]. In Crohn’s disease, the expression of mucin-1 (MUC1) in the inflamed epithelium at the terminal ileus suggests that the mucin cover is insufficient [48]. Paneth cells defend the mucosa by secreting antimicrobial peptide granules, such as a-defensins, and control the composition of the bacterial flora. Paneth cells from patients with Crohn’s disease that harbour mutations in the autophagy gene *ATG16L* have fewer granules, exhibit morphological abnormalities, and are functionally impaired compared with wild-type mice [49].

Further, epithelial cells attach to neighbouring cells through tight-junction proteins such as claudin [50]. In Crohn’s disease, this tight junction becomes leaky because of changes in the expression of tight-junction proteins. This alteration increases cell permeability; luminal antigens access the lamina propria which leads to the accumulation of innate immune cells that produce inflammatory cytokines that activate the adaptive immune system.

Crohn’s disease is characterized by an imbalance between effector T cells and innate regulatory T cells [51]. For example, Th1 and Th17 effector T cells protect the mucosa from bacteria and fungi by secreting IFN-γ, TNF-α, IL-17, and IL-22. Treg cells secrete IL-10 and TGF-β to inhibit the proliferation of dendritic cells and lymphoid cells and induce immune tolerance [52]. These two main opposing phenotypes, Th17 and Treg, originate from CD4+ T cells under stringent negative regulation by the transcription factors RORγt and FOXP3 [53, 54]. Further, the generation of peripherally induced Tregs is influenced by the local microenvironment such as the microbiota and its metabolites, bile acids, and neural stimulation [55–57].

Transforming growth factor (TGF)- β signalling induces Treg differentiation and is required for Th17 cell differentiation. Th17 cells are induced to differentiate from naive CD4^+^ T cells into Th17 cells. This process requires TGF-β and IL-6 signalling, which activates STAT3 to induce the synthesis of RORγt, which is required for the proliferation and survival of Th17 cells. More than 200 genes are associated with susceptibility to IBD, including ulcerative colitis and Crohn's disease [58]. *NOD2* encodes a sensor molecule for bacterial constituent proteins, and variants of *MMP3* contribute to fibrostenosing Crohn’s disease [59]. Furthermore, antibodies to ECM molecules, growth factors, and microbial components may be associated with the development of IBD and intestinal fibrosis.

## Cellular and molecular mechanisms of gastrointestinal fibrosis

**Fig. 2:**
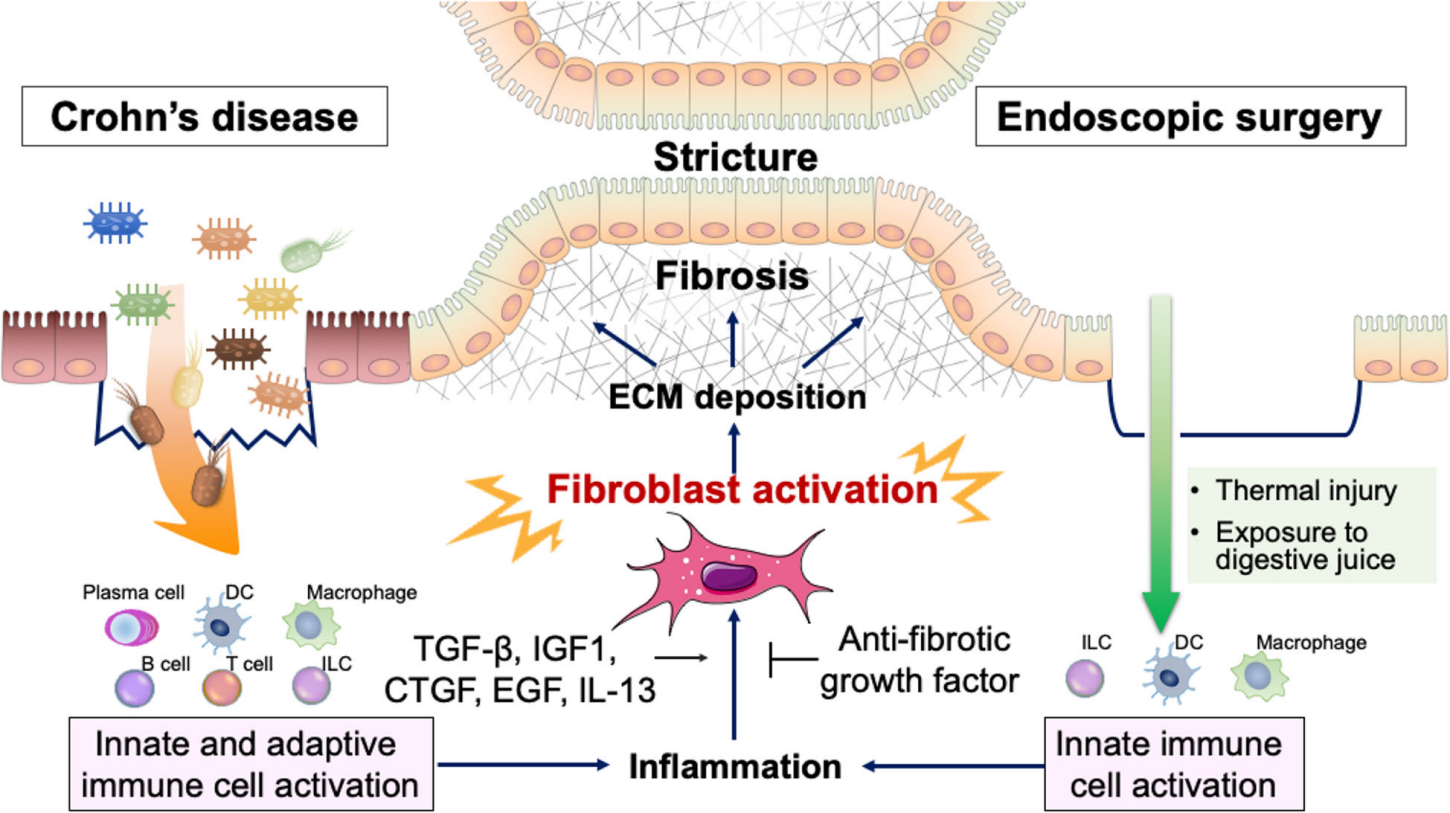
Pathophysiology of GI fibrosis. Crohn’s disease activates innate and adaptive immunity because of genetic abnormalities and the intestinal microflora. Endoscopic treatment activates innate immune cells through thermal injury, exposure to digestive fluid, and submucosal injection, which are strong triggers for ECM-producing cells that cause GI stricture

Fibrosis of the gastrointestinal tract is caused by excessive production of ECM components by activated mesenchymal cells (Fig. 2). After endoscopic treatment, inflammatory cells invade the submucosal layer subsequent to thermal injury and exposure to digestive fluid. In Crohn’s disease, inflammatory cells are induced through the activation of adaptive immunity by intestinal bacteria, as described above. Inflammation potently induces TGF-β signalling, activates ECM-producing cells, and induces tissue fibrosis [60, 61]. ECM-producing cells, which are mainly fibroblasts, comprise a diverse population of cells with diverse origins, including epithelial cells, endothelial cells, astrocytes, and bone marrow-derived stem cells [62].

Fibroblasts are classically characterized through their expression of the cytoskeletal proteins α-SMA, vimentin, CD90 (Thy1), PDGFRα, Sca-1, integrin-α8, CD34, and CD26 (DPP4) [63]. However, recent single-cell omics analyses reveal the functional heterogeneity and tissue specificity of fibroblasts [64–66]. Mesenchymal cells are activated by pathways induced through autocrine and paracrine signalling and microbe-associated and damage-associated molecular patterns. Inhibition of TGF-β signalling leads to prolonged inflammation, because TGF-β, which is induced by inflammation, serves as a mediator of fibrosis and plays a role in the immunomodulation of Treg cells as an inhibitory cytokine [67–69].

Other fibrotic factors include activins, connective tissue growth factor (CTGF), platelet-derived growth factor, insulin-like growth factors (IGF) 1 and 2, epidermal growth factor (EGF), endothelins, and IL-13 which are induced by intense inflammation. However, anti-inflammatory drugs only suppress the generation of inflammatory factors but not fibrotic factors. Thus, evidence indicates that fibrosis is an independent factor of inflammation [70].

Furthermore, the progression of fibrosis is affected by the turnover of ECM components. The generation and degradation of the ECM are balanced by MMPs and MMP inhibitors, and fibrosis occurs when ECM production increases and exceeds its rate of degradation [71, 72]. Recent studies using animal models of fibrosis suggest that pirfenidone, currently approved by the FDA for the treatment of idiopathic pulmonary fibrosis (IPF), an anti-matrix metalloproteinase 9 (MMP9) antibody, OGR1 (pH-sensing ovarian cancer G-protein-coupled receptor 1), and BCL2 inhibitors may prevent fibrosis associated with IBD [73].

## Conclusion and future prospects

Although prophylaxis and treatment have been intensively investigated for preventing and managing gastrointestinal stricture, this condition imposes a great burden on patients and may cause deterioration of their quality of life. Post-endoscopic ulcers cause tissue damage to the submucosa through similar as well as distinct mechanisms responsible for the stricture of Crohn’s disease. The environment of the oesophagus differs from that of the small and large intestines, where there is a small diversity of microbiota, covered with a layer of stratified squamous epithelium, with no immune relay tissues such as those comprising Paneth cells. These environmental tissue factors contribute to pathogenesis and tissue-specific phenotypes of the fibroblasts in the oesophagus and the intestine.

To further dissect tissue specificity of fibroblasts, studies of analogues derived from the skin that share the structural features of the stratified squamous epithelium may help to understand the features of the stroma in the oesophagus that were previously unpredictable. Identification of the tissue-specific roles of fibroblasts in the gastrointestinal tract and identification of common and distinct mechanisms underlying gastrointestinal fibrosis across organs will contribute to our understanding of fibrostenosis under inflammatory and non-inflammatory conditions.

## Data Availability

Not applicable.
